# Gut microbiome alterations and gut barrier dysfunction are associated with host immune homeostasis in COVID-19 patients

**DOI:** 10.1186/s12916-021-02212-0

**Published:** 2022-01-20

**Authors:** Zhonghan Sun, Zhi-Gang Song, Chenglin Liu, Shishang Tan, Shuchun Lin, Jiajun Zhu, Fa-Hui Dai, Jian Gao, Jia-Lei She, Zhendong Mei, Tao Lou, Jiao-Jiao Zheng, Yi Liu, Jiang He, Yuanting Zheng, Chen Ding, Feng Qian, Yan Zheng, Yan-Mei Chen

**Affiliations:** 1 State Key Laboratory of Genetic Engineering, School of Life Sciences and Human Phenome Institute, Fudan University, Shanghai, China; 2 Shanghai Public Health Clinical Center, State Key Laboratory of Genetic Engineering, School of Life Sciences and Human Phenome Institute, Fudan University, Shanghai, China

**Keywords:** COVID-19, SARS-CoV-2, Microbiome, Metaproteomic, Gut barrier, Immune homeostasis

## Abstract

**Background:**

COVID-19 is an infectious disease characterized by multiple respiratory and extrapulmonary manifestations, including gastrointestinal symptoms. Although recent studies have linked gut microbiota to infectious diseases such as influenza, little is known about the role of the gut microbiota in COVID-19 pathophysiology.

**Methods:**

To better understand the host-gut microbiota interactions in COVID-19, we characterized the gut microbial community and gut barrier function using metagenomic and metaproteomic approaches in 63 COVID-19 patients and 8 non-infected controls. Both immunohematological parameters and transcriptional profiles were measured to reflect the immune response in COVID-19 patients.

**Results:**

Altered gut microbial composition was observed in COVID-19 patients, which was characterized by decreased commensal species and increased opportunistic pathogenic species. Severe illness was associated with higher abundance of four microbial species (i.e., *Burkholderia contaminans*, *Bacteroides nordii*, *Bifidobacterium longum*, and *Blautia* sp. CAG 257), six microbial pathways (e.g., glycolysis and fermentation), and 10 virulence genes. These severity-related microbial features were further associated with host immune response. For example, the abundance of *Bu. contaminans* was associated with higher levels of inflammation biomarkers and lower levels of immune cells. Furthermore, human-origin proteins identified from both blood and fecal samples suggested gut barrier dysfunction in COVID-19 patients. The circulating levels of lipopolysaccharide-binding protein increased in patients with severe illness and were associated with circulating inflammation biomarkers and immune cells. Besides, proteins of disease-related bacteria (e.g., *B. longum*) were detectable in blood samples from patients.

**Conclusions:**

Our results suggest that the dysbiosis of the gut microbiome and the dysfunction of the gut barrier might play a role in the pathophysiology of COVID-19 by affecting host immune homeostasis.

**Supplementary Information:**

The online version contains supplementary material available at 10.1186/s12916-021-02212-0.

## Background

Coronavirus disease 2019 (COVID-19), caused by a novel beta-coronavirus (severe acute respiratory syndrome coronavirus 2 (SARS-CoV-2)), has been a global pandemic and caused more than five million deaths worldwide until November of 2021 [1]. The primary symptoms of COVID-19 are demonstrated in the respiratory system, and extrapulmonary manifestations including gastrointestinal (GI) symptoms, thrombotic complications, and myocardial dysfunction are common [2]. SARS-CoV-2 could invade human cells via the angiotensin-converting enzyme 2 (ACE2) receptor, which is highly expressed in intestines and plays an important role in maintaining gut health [3–5]. The infection of SARS-CoV-2 could impair the normal expression of ACE2, which might result in several adverse outcomes, including GI symptoms as well as the dysbiosis of gut microbiota [6]. Reports from multiple regions of the world showed that 15% to 69% of COVID-19 patients had at least one GI symptom [7–10].

The microbial communities that reside in the human gut could maintain host homeostasis by providing essential functions, including immunomodulation, nutrient metabolism, and structural protection against pathogenic microorganisms [11–13]. Altered gut microbiota was observed among patients with a wide range of infectious diseases, including influenza and other respiratory viral infections [14–17]. Recent studies also described the alterations in the gut microbial composition of COVID-19 patients, characterized by enrichment of opportunistic pathogens and depletion of beneficial commensals [18–20]. In addition, three bacterial members from the *Firmicutes* phylum were positively and two beneficial species, *Alistipes onderdonkii* and *Faecalibacterium prausnitzii* were inversely associated with COVID-19 severity [21]. However, the potential mechanism underlying the associations between the gut microbiome and COVID-19 severity remains to be explored.

SARS-CoV-2 infection induces the host immune responses to eliminate the virus, and previous evidence suggested that aberrant immune responses were responsible for adverse outcomes and possibly other inflammations beyond COVID-19 [22, 23]. The GI tract is the largest immunological organ in the human body and its resident microbiota are known to modulate host immune responses [24, 25]. According to a prospective study, the gut microbial composition was correlated with the increase of inflammation markers, including interleukin (IL)-10, tumor necrosis factor-α, and C-reactive protein (CRP) in COVID-19 patients [21]. Nevertheless, data revealing the global relations between the gut microbiome and host systemic immune response in COVID-19 are still limited.

The microbiota-host immune interactions could be mediated by other host factors such as gut barrier function. Intestinal epithelial cells provide a physical and biochemical barrier that segregates host tissue and bacteria to maintain intestinal homeostasis [26]. Both virus infection and altered gut microbiota could disturb the normal function of the gut barrier and lead to a leaky gut with enhanced gut permeability [27], which aggravates over-activation of the host immune response [28, 29]. Thus far, no study has characterized the role of gut barrier dysfunction in the relationship between gut microbes and host immune homeostasis in COVID-19 patients, which may deepen our understanding of COVID-19 pathophysiology.

To better understand the role of gut microbiota in COVID-19 pathogenesis, we characterized the gut microbiota and gut barrier function among 63 COVID-19 patients and 8 uninfected controls through metagenomic and metaproteomic approaches and estimated the associations of gut microbiota with disease severity as well as host systemic immune responses.

## Methods

### Study population

According to local emergency regulations, all adult COVID-19 patients in Shanghai city were admitted to the Shanghai Public Health Clinical Center. All patients that were treated at the center between January 31 and April 7, 2020, were invited to participate in the study. Uninfected volunteer hospital staff were recruited as controls at the same time. None of the controls had any recent infection episodes, antibiotics use, probiotics use, or any medication within the 2 weeks prior, or ever received chemotherapy treatment. Eventually, a total of 63 patients (39 mild cases and 24 severe cases) and 8 controls were enrolled in this study. Data regarding demographics, medical measurements, and antibiotic use during hospitalization were extracted from the electronic medical records (Additional file 2: Table S1).

### Sample collection and SARS-CoV-2 detection

A total of 106 stool samples were collected serially from COVID-19 patients and uninfected controls by professional healthcare workers over a 5-week period. All samples were collected using sterile containers and were stored at -80 °C immediately until processing. Notably, each included patient provided one fecal sample at admission. For patients with severe COVID-19, fecal samples were further collected weekly during their hospital stay. All these samples were classified according to providers’ disease severity status (mild vs. severe condition) when they were collected (Additional file 2: Table S2).

For the detection and quantification of SARS-CoV-2, viral RNA was extracted using a nucleic acid isolation kit with magnetic beads (Jiangsu Bioperfectus technologies) following the manufacturer’s instructions. The RNA extractions were then subject to SARS-CoV-2 detection by quantitative real-time RT-PCR as previously described [30]. For positive samples, viral load was determined using digital RT-PCR as we previously reported [31].

### Laboratory measurement

Testing for blood cells, complement, inflammation biomarkers, kidney function biomarkers, renal function biomarkers, and coagulation function biomarkers were performed using a hematology analyzer (Hematology Analyzer XN-1000, Sysmex). Full lymphocyte subsets and cytokines were analyzed using FACSCanto II flow cytometer (BD Biosciences) with FlowJo V10.7.2 software (BD Biosciences).

### Shotgun metagenomic sequencing

Total bacterial genomic DNA was extracted using the nucleic acid isolation kit (BioPerfectus technologies company). Metagenomic DNA samples were normalized to a concentration of 1 ng/μl to prepare Illumina sequencing libraries using the Tn5 DNA Library Prep Kit for Illumina (APExBIO), according to the workflow described elsewhere [32]. Whole-genome shotgun sequencing of fecal samples was carried out on the Illumina Novaseq6000 platform (PE150; paired-end; insert size, 350 bp; read length, 150 bp).

The quality control of whole-genome shotgun sequencing data was performed by KneadData (version 0.7.2), which contains Trimmomatic (version 0.33) and Bowtie2 (version 2.3.4.3) [33, 34]. After quality control, we obtained on average 45.1 million high-quality reads (~ 6.4GB) per sample. The taxonomic profiles of metagenomics were determined by MetaPhlan (version 3.0.3) [35]. Only 177 microbial species that presented in more than 10% of total samples (20 samples) were included in our analysis. The MetaCyc pathways were determined by HUMAnN (version 3.0.0.alpha.3) and only those presented with the top 75% relative abundance were included in the downstream analysis [35, 36].

Analysis of bacterial virulence-associated genes in the metagenomes was performed using a custom virulence factor database generated from UniRef90 and VFDB by shortBRED (version 0.9.5) [37–39]. The abundances of virulence genes were normalized to the number of clean reads per sample to get relative abundance. Virulence genes that presented in more than 10% of total samples were included in the downstream analysis.

### Metaproteomics and proteomics measurement

Each fecal sample for metaproteome measurements (~ 350 mg for each sample) was solubilized in 1 mL SDS lysis buffer [4% w/v SDS, 100 mM Tris.HCl (pH 8.0), 1 mM PMSF] and incubated for 10 min. To inactivate the virus, the mixture was put in the metal bath at 100 °C for 15 min and the water bath at 56 °C for 30 min. Then, we centrifuged it at 21,000×*g* for 60 min. Take the supernatant as crude protein extract and wash it with ice-cold acetone to remove lipids and excess SDS. The protein precipitates were resolubilized via sonication in 500 μl of 8 M urea in 100 mM Tris-HCl (pH 8.0) and quantified using a bicinchoninic acid-based protein assay kit. Samples were normalized for concentration by diluting the crude protein to 2 mg/μl with urea buffer [8 M urea, 100 mM Tris.HCl (pH 8.0)]. Then these samples were transferred to new EP tubes and diluted further with CaCl_2_ buffer [10 mM CaCl_2_, 100 mM Tris.HCl (pH 8.0)] to a final urea concentration below 4 M. After that, samples were reduced by incubation with DTT at a final concentration of 10 mM for 1 h at room temperature. Then samples were initially purified by SDS-PAGE and stored at 4 °C. The workflow of blood samples processing for proteomics measurement was described elsewhere [31]. Processed samples were measured using LC-MS instrumentation consisting of an EASY-nLC 1200 ultra-high-pressure system (Thermo Fisher Scientific) coupled via a nano-electrospray ion source to Fusion Lumos Orbitrap (Thermo Fisher Scientific).

Peptide spectrum mapping and quantitation of proteins in fecal samples were performed using the MetaProteomeAnalyzer with default settings [40]. Human and nonhuman proteins were scaled to the sum of the total human or nonhuman peptide counts, respectively. We applied the same metaproteome pipeline as fecal data to identify bacterial proteins in blood samples. For the searching setting, a maximum of two missed cleavages was allowed. The protein expression of a bacterial species in a person was calculated as the sum of peptide count of this species. The detailed information about peptide identification and protein quantification of proteins in blood samples was described elsewhere [31].

### Whole blood transcriptomic data selection and processing

Twenty-nine patients enrolled in this study were analyzed in our previous study and had whole blood transcriptomic data, and the transcriptomic results were reported in detail in our previous study [31]. The RNA-seq data of these patients were collected, processed, and filtered as described previously [31]. GSEA (Gene Set Enrichment Analyses) was performed to identify significantly enriched functional classes of gene sets correlated with blood transcription modules, and the activity of each module was calculated as the mean expression value of member genes [41].

### Statistical analyses

The distributions of basic characteristics of the study population according to COVID-19 status were compared using Student’s *t*-test or Wilcoxon rank-sum test for continuous variables and *χ*^2^ test or Fisher’s exact test for categorical variables. Alpha (Simpson and Shannon indexes) and beta diversities (Unweighted Unifrac distance) metrics were calculated based on species relative abundance identified from whole-genome shotgun sequencing. The significance of the microbial diversity difference between groups was assessed by the Wilcoxon rank-sum test and permutational multivariate analysis of variance (PERMANOVA). The variation of microbial diversity explained by host factors was calculated using PERMANOVA with a permutation of 9 999 times via R package vegan (version 2.5-6). LDA Effect Size (LEfSe) analysis was performed to define COVID19-related microbial features (species and Metacyc pathways). Mixed linear regression was performed to estimate the pairwise associations between differential microbial features and clinical phenotypes with individual variance as a random effect. Age and sex were adjusted for the above regression analyses. P values were adjusted for multiple comparisons using the Benjamini-Hochberg method (*q* value). A *P* value of < 0.05 or *q* value of < 0.25 were considered to be statistically significant. To reduce the impact of outliers and deviations from normality, all microbial features, cardiometabolic phenotypes, and metabolites were transformed using inverse rank-sum transformation before analysis. All the data analyses were conducted in R (version 3.6.1).

## Results

### Patient cohort and sample collection

We recruited 63 clinically diagnosed and laboratory-confirmed COVID-19 patients, who were hospitalized at Shanghai Public Health Clinical Center from January 31 to April 7, 2020, and 8 non-infected volunteers as controls. The COVID-19 patients included 39 males and 24 females with ages ranging from 12 to 83 years (median, 45 years), while uninfected volunteers contained 3 males and 5 females with ages ranging from 23 to 56 years (median, 37 years). The length of hospital stay of COVID-19 patients ranged from 8 to 75 days (median, 26 days). The clinical features at baseline and treatment during hospitalization were described in Table 1 and the detailed information of each patient was shown in Table S1. In line with previous reports [18], COVID-19 patients with severe condition were more likely to be older, present respiratory and gastrointestinal symptoms, and have higher circulating levels of inflammation biomarkers (e.g., CRP), immune cells (e.g., lymphocyte, CD4+, CD8+), and cytokines (e.g., IL-6, IL-10) (all *P* < 0.05, Table 1). These biomarkers were also associated with the progression of COVID-19 (Additional file 1: Fig. S1). Among the patients with severe condition, about 58% were intubated and 33% were supported with extracorporeal membrane oxygenation during their hospitalization.

**Table 1:** The basic information and clinical characteristics of COVID-19 patients

	Total *N*=63	Mild *N*=39	Severe *N*=24	*P* value
Age, years	48.0 ± 21.2	40.1 ± 19.8	61.0 ± 16.8	< 0.001
Male (%)	39 (61.9)	20 (51.3)	19 (79.2)	0.05
Length of hospital stay, days	26.1 ± 18.8	15.8 ± 4.1	42.8 ± 21.5	< 0.001
Viral load, Log10(copies/μl)	3.7 ± 1.6	3.7 ± 1.7	3.8 ± 1.5	0.16
Symptoms at admission (%)				
Fever	41 (65.1)	19 (48.7)	22 (91.7)	< 0.001
Chest tightness	20 (31.7)	7 (17.9)	13 (54.2)	0.005
Cough	33 (52.4)	21 (53.8)	12 (50.0)	0.80
Sputum	25 (39.7)	12 (30.8)	13 (54.2)	0.11
Diarrhea	9 (14.3)	3 (7.7)	6 (25.0)	0.07
Death (%)	5 (7.9)	0	5 (20.8)	0.01
Laboratory measurement				
C-reactive protein, mg/L	13.1 (1.5, 58.6)	4.4 (1.5, 11.7)	61.6 (42.8, 117.5)	< 0.001
Lymphocyte count, × 10^9^/L	0.8 (0.6, 1.2)	1.2 (0.9, 1.4)	0.5 (0.4, 0.7)	< 0.001
Neutrophil cell count, ×10^9^/L	6.1 (4.3, 9.3)	5.1 (4.1, 6.2)	8.7 (5.1, 12.7)	0.02
White blood cell count, ×10^9^/L	4.4 (3.1, 7.6)	3.1 (2.5, 4.5)	7.2 (4.3, 11.7)	0.001
CD4+, cell/μl	431 (253, 725)	589 (422.5, 924)	158 (97.8, 329.8)	< 0.001
CD8+, cell/μl	223 (130.5, 424.5)	353 (208.5, 500)	104.5 (48.8, 163.2)	< 0.001
IL-6, pg/mL	1.4 (0, 10.5)	0 (0, 1.6)	20.3 (6.6, 60.9)	< 0.001
IL-10, peg/mL	0.4 (0.3, 0.9)	0.3 (0.2, 0.5)	1.1 (0.5, 2.7)	< 0.001
Lactate dehydrogenase, U/L	336 (207.8, 508.2)	209 (179, 257)	512 (455.5, 605.5)	< 0.001
Antivirals during hospitalization (%)				
Lopinavir/ritonavir	10 (15.9)	2 (5.1)	8 (33.3)	0.005
Arborol	15 (23.8)	4 (10.3)	11 (45.8)	0.002
Hydroxychloroquine	35 (55.6)	26 (66.7)	9 (37.5)	0.04
Interferon	20 (31.7)	15 (38.5)	5 (20.8)	0.17
Antibiotics during hospitalization (%)				
Moxifloxacin	13 (20.6)	3 (7.7)	10 (41.7)	0.003
Other antibiotics	7 (11.1)	1 (2.6)	6 (25.0)	0.01
Tracheal intubation (%)	14 (22.2)	0 (0.0)	14 (58.3)	< 0.001
Extracorporeal membrane oxygenator (%)	8 (12.7)	0 (0.0)	8 (33.3)	< 0.001

^1^For patients with mild disease, the laboratory measurements presented were the first measurement after admission; for patients with severe disease, the clinical characteristics presented were the first measurement after being diagnosed as severe condition

^2^Data were shown as mean ± SD or median (lower quartile, upper quartile) for continuous variable and number (%) for the categorical variable. Group differences were calculated using Student’s *t* test, Wilcoxon rank-sum test, *χ*^2^ test, or Fisher’s exact test

### Gut microbiome in COVID-19 patients

**Fig. 1:**
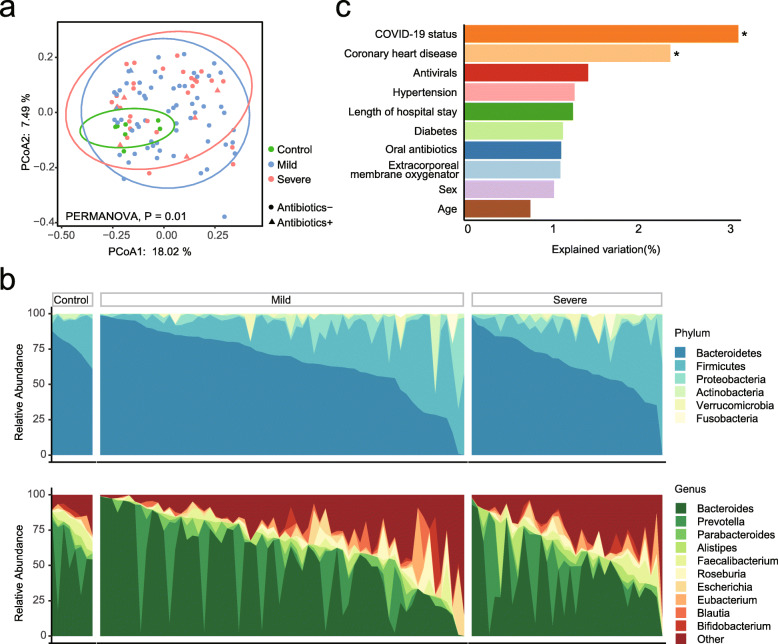
Alterations in gut microbiome composition of COVID-19 patients. **a** The composition of gut microbiota significantly altered in COVID-19 patients. The microbial composition was represented by the β-diversity based on unweighted Unifrac distance. **b** The phylum (up) and genus (down) distribution of the gut microbiota of COVID-19 patients and non-COVID-19 controls. **c** The microbial variation explained by medication and basic characteristics. Asterisk (*) represents significant associations by PERMANOVA

To better understand the effect of SARS-CoV-2 infection on the gut microbiome, we studied the gut microbial communities of COVID-19 patients, by using metagenomic sequencing in 106 fecal samples collected serially from COVID-19 patients and non-infected controls (Additional file 1: Fig. S2 and Additional file 2: Table S2). Although the α-diversity of the fecal microbiome showed no significant difference (*P* > 0.05, Additional file 1: Fig. S3), distinct microbial composition was observed in COVID-19 patients compared with non-infected controls (*P* = 0.01, PERMANOVA, Fig. 1a, b). The SARS-CoV-2 infection explained 3.2%, and CHD explained 2% of the total variations in the microbial composition (*P* < 0.05, PERMANOVA, Fig. 1c), whereas comorbidities of hypertension and diabetes, length of hospital stay, antibiotic/antiviral treatment, or other treatments (i.e., extracorporeal membrane oxygenator and trachea intubation) during hospitalization did not impact microbial composition significantly (all *P* > 0.05).

At phylum and genus levels, a higher abundance of phylum *Verrucomicrobia* and lower abundances of three common genera (*Faecalibacterium*, *Dialister*, and *Lachnospira*) in phylum *Firmicutes* were observed in COVID-19 patients (*P* < 0.05, LDA score > 2, LEfSe, Fig. 1c and Additional file 2: Table S3). At the species level, the abundance in patients was lower for 19 species and higher for 8 species when compared with controls [*P* < 0.05, LDA score > 2, LEfSe, Additional file 2: Table S3]. Further adjustment of age, sex, and history of CHD did not change the results materially, and all these 27 COVID-19-related species reminded significantly associated with COVID-19 (all *P*< 0.05, Additional file 2: Table S3). Similar to previous findings [21], the microbial alteration in COVID-19 patients was characterized by the depletion of potential beneficial microbiota, such as *Faecalibacterium prausnitzii* and *Bifidobacterium pseudocatenulatum*, and fermentative bacteria, such as *Eubacterium eligens*, *Bacteroides eggerthii*, *Alistipes shahii*, *Lawsonibacter asaccharolyticus*, and *Bacteroides cellulosilyticus*. These commensal species are known to help maintain gut function and immune homeostasis [42]. Importantly, the abundance for some opportunistic pathogens (e.g., *Bacteroides ovatus*, *Acinetobacter bereziniae*, and *Clostridium innocuum*) were also enriched in COVID-19 patients.

### Variations in the gut microbiome between patients with mild and severe illness

**Fig. 2:**
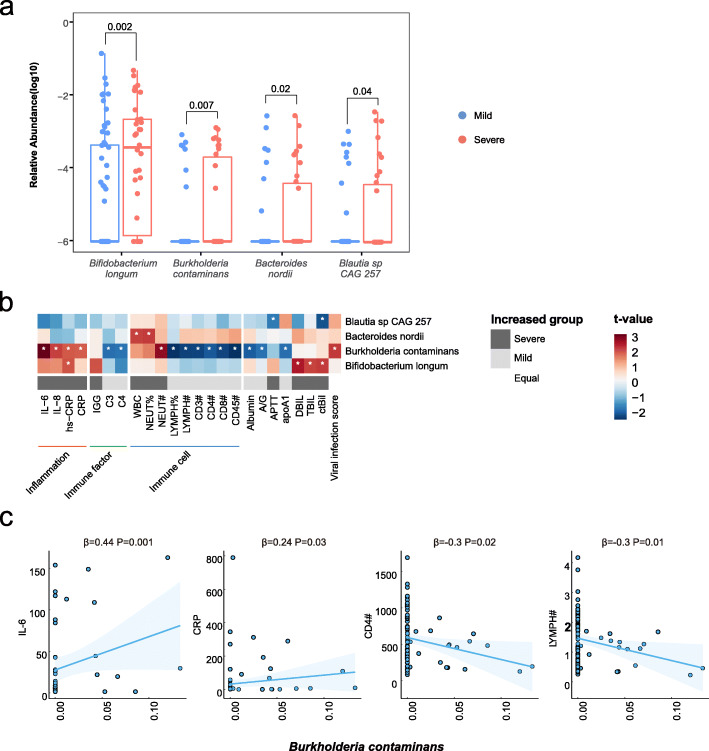
Associations of gut microbial species with COVID-19 severity and host immune response. **a** Relative abundances of the 4 different species in patients with severe condition or mild condition at the criteria of *P* < 0.05 and LDA > 2 by LEfSe. The numbers represent *P*-value of the Wilcoxon rank-sum test. **b** Associations of differential microbial species with clinical traits with adjustment for age and sex. Red bars indicate positive associations, and blue bars indicate negative associations. White asterisks indicate associations with *P* < 0.05. The color key indicates the association strength and direction in terms of the t-value. The gray bar shows in which group the corresponding indicator is higher. The bottom color bar shows the classifications of clinical traits. The percent sign (%) represents the percentage, and the pound sign (#) represents the count value of the corresponding immune cells. **c** The associations between the relative abundance of *Burkholderia contaminans* and circulating levels of IL-6, CRP, and counts of CD4+ T cell and total lymphocyte

To understand whether the gut microbiome was associated with COVID-19 severity, we further compared the gut microbiome between patients with mild illness and those with severe illness. Although the microbial composition was similar between the two groups (*P* > 0.05, Additional file 1: Fig. S3), four microbial species had significantly higher abundances in severe COVID-19 patients. Compared with patients with mild disease, the average abundance in patients with severe COVID-19 increased by 117% for *Bacteroides nordii* (mean abundance 0.01 in mild patients vs. 0.02 in severe patients), 327% for *Burkholderia contaminans* (0.004 vs. 0.02), 30% for *Bifidobacterium longum* (0.41 vs. 0.53), and 569% for *Blautia* sp. CAG 257 (0.004 vs. 0.03) (all *P* < 0.05, LEfSe, Fig. 2a). In addition, the abundances of these microbes in some patients tended to increase over the deterioration period and decrease over the alleviation period (Additional file 1: Figs. S4–S7).

As the aberrant immune responses and multi-organ injuries were responsible for the poor prognosis of COVID-19, we attempted to explore the interplay of the gut microbiome with specific blood biomarkers, which reflected inflammation, immunopathology, and multi-organ damage. Consequently, mixed linear regression analyses revealed 25 significant associations between four microbial species and blood biomarkers (*q* < 0.05, Additional file 2: Table S4). Notably, opportunistic pathogens *Ba. nordii* and *Bu. contaminans* were responsible for the majority of such associations, especially with the immune biomarkers. *Ba. nordii* was positively associated with the total count of white blood cell (*β* coefficient = 0.29) and the percentage of neutrophils (*β* coefficient = 0.27). *Bu. contaminans* was associated with lower circulating levels of total lymphocytes counts (*β* coefficient = − 0.33), CD3+ T cells counts (*β* coefficient = − 0.31), CD4+ T cells counts (*β* coefficient = − 0.30), and complements C3 (*β* coefficient = − 0.31), C4 (*β* coefficient = − 0.33), as well as higher circulating levels of hs-CRP (*β* coefficient = 0.24) and IL-6 (*β* coefficient = 0.44) (Fig. 2b, c). Furthermore, *Bu. contaminans* was also inversely related to T cell-response, indicated by a gene set from the whole blood transcriptomic data (Additional file 1: Fig. S8) [31]. In sum, these data suggest an association between microbial variations, aberrant immune response, and COVID-19 severity.

### Effect of oral antibiotics on the gut microbiome in COVID-19 patients

To examine whether the gut microbial variations in COVID-19 patients were significantly influenced by antibiotic intervention, we analyzed the gut microbiome in severe samples with (n=7) or without (n=26) oral antibiotics use. No significant alteration was observed in the microbial composition between the two groups (Additional file 1: Fig. S9). The abundances were significantly increased for five microbial species (i.e., *Acinetobacter guillouiae*, *Megamonas hypermegale*, *Megamonas funiformis*, *Sutterella parvirubra*, *Serratia liquefaciens*) in the patients with antibiotics use, while decreased for the bacteria *Ruminococcus bicirculans* (*P* < 0.05, Additional file 2: Table S5). Statistical analysis was not applicable for the mild illness group as only one patient received oral antibiotics. However, for the microbial species which varied significantly between COVID-19 patients and controls as well as between patients with mild and severe disease, their relative abundances were not significantly different in patients with and without antibiotics use (Additional file 2: Table S5). Additionally, after excluding patients with oral antibiotics (*n* = 9), 24 out of the 26 disease-related species remained differently enriched between COVID-19 patients and controls (*P* < 0.05, LDA score > 2, LEfSe, Additional file 2: Table S6). These results indicated the effects of SARS-CoV-2 infection on the gut microbiome were not dependent on antibiotics use in our study.

### Microbial functions were associated with COVID-19 severity and immune homeostasis

**Fig. 3:**
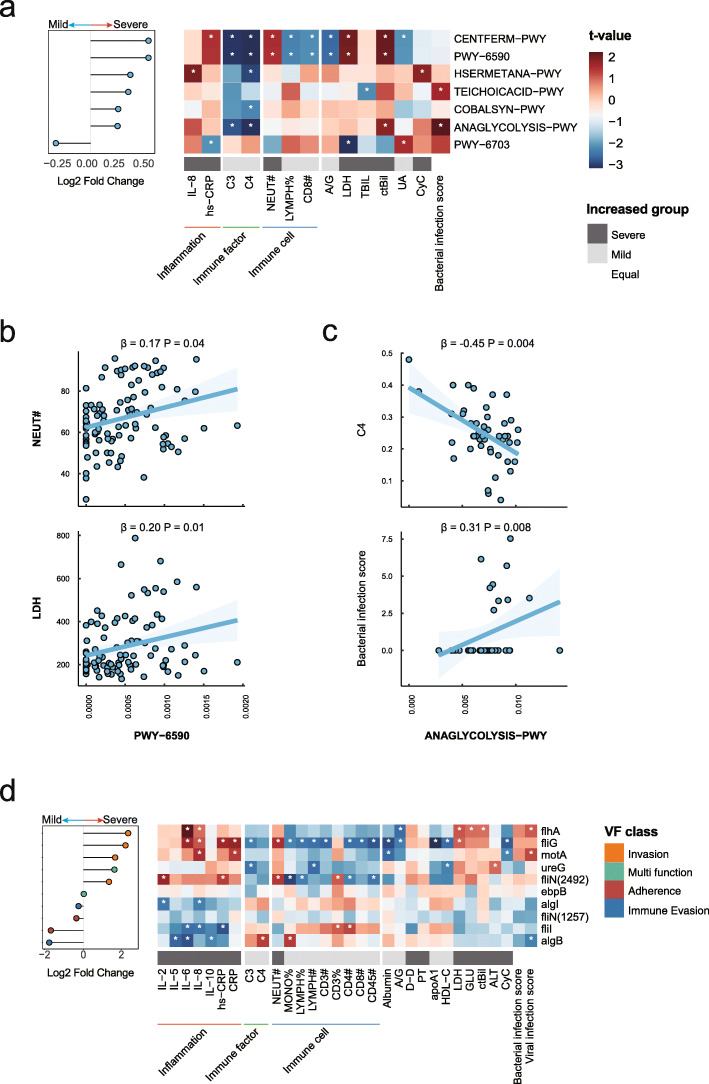
Relationships of microbial functional potentials with COVID-19 severity and host immune response. **a** Seven COVID-19 severity-related microbial pathways and their associations with clinical traits. Red bars indicate positive associations, and blue bars indicate negative associations. White asterisks indicate associations with *P* < 0.05. The color key indicates the association strength and direction in terms of the t value. The gray bar shows in which group the corresponding indicator is more abundant. The percent sign (%) represents the percentage, and the pound sign (#) represents the count value of the corresponding immune cells. **b** The associations of the relative abundance of carbohydrate pathway (PWY-6590) with levels of lactate dehydrogenase and counts of neutrophils. **c** The associations of the relative abundance of glycolysis pathway (ANAGLYCOLYSIS-PWY) with levels of complement C4 and bacterial infection score. **d** COVID-19 severity-related virulence genes and their associations with clinical traits. The VFs-color bar shows the classification of VFs

We next investigated the COVID-19 severity in relation to the functional potentials of the gut microbial communities. From the fecal samples collected in this study, we identified a total of 386 microbial pathways based on the MetaCyc database [36]. Among these pathways, preQ0 biosynthesis (PWY-6703) was enriched in patients with mild disease and six other pathways were more abundant in patients with severe condition, i.e., glycolysis, fermentation, methionine biosynthesis, vitamin B12 biosynthesis, and teichoic acid biosynthesis (*P* < 0.05, LDA score > 2, LEfSe, Fig. 3a). We further assessed the associations between these microbial pathways and immunity biomarkers in COVID-19 patients. As a result, a total of 33 associations were observed between severity-related pathways and host immune response indices (*P* < 0.05, Fig. 3a and Additional file 2: Table S7). For instance, the abundances of microbial fermentation pathways (PWY-6590 and CENTFERM-PWY) were positively associated with the circulating levels of neutrophils count and lactate dehydrogenase while negatively associated with the percentage of lymphocytes (all *P* < 0.05, Fig. 3b). In addition, the glycolysis pathway (ANAGLYCOLYSIS-PWY) had inverse associations with circulating levels of complements C3 and C4, and a positive association with the bacterial infection score generated from whole blood transcriptomic data (all *P* < 0.05, Fig. 3c).

In addition to the microbial metabolic dysfunction, bacterial virulence factors may also influence immune homeostasis [43]. We thus identified the microbial genes encoding virulence factors by sequence alignment approach based on Virulence Factor Database [38]. Compared with those in COVID-19 patients with mild disease, a total of 10 virulence genes had significantly higher abundances (*P* < 0.05, Wilcoxon rank-sum test, Fig. 3d) in patients with severe disease. These enriched virulence genes could contribute to the pathogenic potential of bacteria through various mechanisms, such as those that could increase bacteria’s ability to invade human tissue (fliN, flhA, fliG, and motA), to escape the host immune response (algB and algI), and to colonize (ebpB, fliI, fliN, and ureG). Notably, the virulence genes related to bacteria’s invasion ability were positively associated with the patients’ circulating levels of inflammatory biomarkers (IL-6, IL-8, and hs-CRP) and negatively associated with the circulating absolute counts of CD3+ and CD4+ T cells (*P* < 0.05, Fig. 3d and Additional file 2: Table S8).

Combined, these data suggested that the microbial metabolic function, especially glucose metabolism, and enriched virulence genes might mediate the associations between the gut microbiome and the aberrant immune response in COVID-19.

### Gut barrier dysfunction in COVID-19 patients

**Fig. 4:**
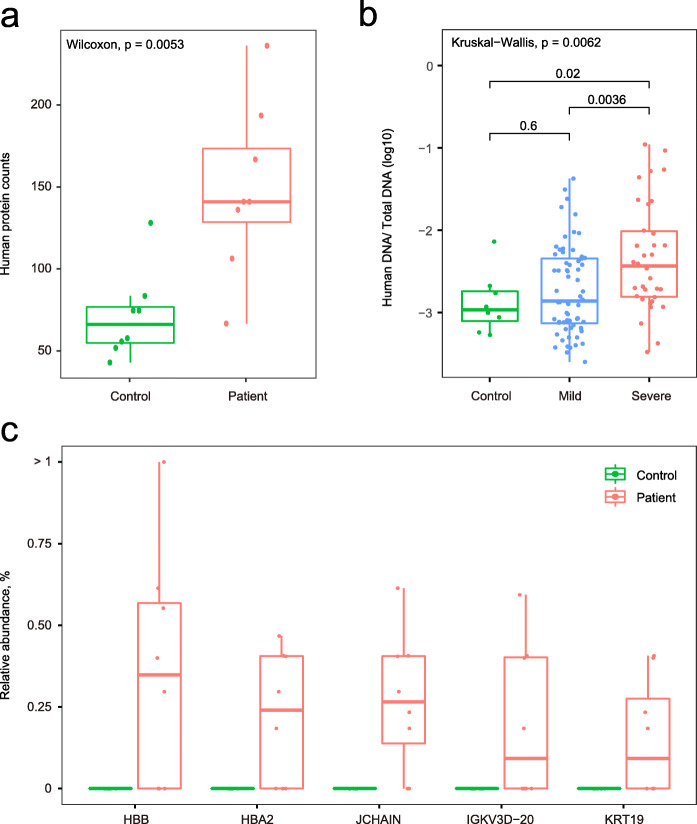
Gut barrier dysfunction in COVID-19 patients. **a** Number of human proteins detected in fecal samples from COVID-19 patients and controls. **b** Human-to-all DNA ratio detected in fecal samples from COVID-19 patients and controls. **c** The relative abundances of candidate human fecal proteins related to gastrointestinal damage

Both intestinal infection of SARS-CoV-2 and gut microbial dysbiosis could result in gut barrier dysfunction [27]. To assess the extent of gut barrier dysfunction in COVID-19 patients, metaproteome profiles were characterized using fecal samples from 16 patients and controls (Additional file 1: Fig. S10). A total of 4094 proteins (21,037 peptides) were identified. Of which, 650 proteins (675 peptides) were annotated to human, and 1585 proteins (16,571 peptides) were annotated to 631 known microbial species (Additional file 1: Fig. S11). The human protein richness in fecal samples was higher in patients compared to that in controls (*P* = 0.005, Wilcoxon rank-sum test, Fig. 4a), and this was corroborated by the increased ratio of human DNA in fecal samples of patients with severe illness (severe vs. non-infected *P* = 0.02, severe vs. mild *P* = 0.003, Wilcoxon rank-sum test, Fig. 4b), suggesting a potential intestinal epithelial damage in patients with more severe disease. Remarkably, of the 40 differential abundant human proteins identified from fecal samples, 34 proteins were upregulated in COVID-19 patients (*P* < 0.05, Additional file 1: Fig. S12). Some of these differentially enriched proteins reflected the intestinal infection of SARS-CoV-2 and intestinal injury. For example, the protein components of human immunoglobulin (JCHAIN and IGKV3D-20) and human hemoglobin proteins (HBB and HBA) were upregulated in COVID-19 patients, suggesting an enhanced immune response and potential bleeding in their intestines. In addition, a protein of cell skeleton and barrier (KRT19) was also enriched in fecal samples of patients (Fig. 4c), providing a clue of gastrointestinal cell damage.

To further explore the gut barrier dysfunction in COVID-19 patients, lipopolysaccharide-binding protein (LBP), a biomarker of gut barrier dysfunction [44], was measured using proteomic approaches with plasma samples from COVID-19 patients (*n*=148) [31]. Compared with patients with mild condition, the circulating levels of LBP increased significantly in those with severe condition (*P* < 0.05, Additional file 1: Fig. S13). Furthermore, the circulating level of LBP was associated with inflammation biomarkers (hs-CRP, CRP, IL-5, IL-6, and IL-8), immune cells (percentage of lymphocytes and percentage of neutrophils), and lactate dehydrogenase (all *P* < 0.05, Additional file 2: Table S9).

The gut barrier dysfunction could increase the microbial translocation into blood [45], which plays a key role in activating the systemic immune response [46]. We therefore measured the bacterial proteins in blood samples in our COVID-19 patients. As a result, a total of 73 microbial proteins were identified among all the collected samples, annotated 26 microbial genus and 18 microbial species (Additional file 2: Table S10). Notably, some proteins that were only detected in the plasma of COVID-19 patients belong to bacteria that were enriched in the fecal samples from COVID-19 patients, including genus *Burkholderia*, genus *Pseudomonas*, and species *B. longum* (Additional file 1: Fig. S14). However, the detection rate of bacterial proteins in the blood sample was too low to perform statistical analysis. In sum, these results suggested that the gut barrier dysfunction might be a mediator in the interactions between the gut microbiome and immune homeostasis in COVID-19.

## Discussion

It has been known that the gut system is actively involved in COVID-19 pathophysiology for the high expression of ACE2, which is the receptor of SARS-CoV-2 [3, 4]. The intestinal infection of SARS-CoV-2 could lead to the disruption of the intestinal homeostasis and the host immune homeostasis, which were responsible for the adverse outcomes of COVID-19. In the current study, we observed a significant change in the composition of gut microbiota of COVID-19 patients compared with controls and identified several microbial features at both taxonomic and functional levels associated with COVID-19 severity and host immune responses. Besides, through an integrative analysis of multi-omics data, we found that gut barrier dysfunction might play a role in the crosstalk between gut microbes and host immune homeostasis in COVID-19 patients.

Our results echo the findings from previous studies, reporting the microbial species alterations that were associated with COVID-19 status [19, 21]. In our study, several commensal species, such as *Ba. uniformis*, *F. prausnitzii*, and *Bi. pseudocatenulatum*, as well as fermentative species, including *E. eligens* and *Ba. eggerthii*, were depleted in the gut microbiota of COVID-19 patients. Of which, *F. prausnitzii* were also found depleted in COVID-19 patients in another Chinese study [21]. These commensal species could maintain the physical separation between the microorganism and the host and prevent the invasion of pathogens through multiple approaches, including secreting anti-microbial peptides and SCFAs [47, 48].

The decrease of commensal species might disturb the normal function of the gut barrier and lead to a leaky gut with enhanced gut permeability [27]. In the current study, we observed the gut barrier dysfunction characterized by metaproteomic alterations in COVID-19 patients. The increased intestinal permeability was further supported by higher circulating levels of LBP and the detection of bacterial proteins in blood samples in our patients. Lipopolysaccharide is the major outer membrane pathogen-associated molecular pattern of Gram-negative bacteria which can cause an acute inflammatory response by triggering the release of a vast number of inflammatory cytokines [49]. The leaky gut might promote the transportation of microbes or endotoxins like lipopolysaccharide from the intestine into the blood, which could lead to the immune homeostasis disturbance of COVID-19 patients. Together, these lines of evidence suggested that the alterations of the gut microbiome were associated with SARS-CoV-2 infection and such associations might be mediated by the gut barrier dysfunction in COVID-19 patients.

The dysbiosis of the gut microbiome and dysfunction of the gut barrier could influence the balance between gut microbiota and host, resulting in a worsened inflammation-induced injury [25]. Over-reaction of the human immune response was the major reason for the poor prognosis of COVID-19 [22, 50]. In our study, the COVID-19 patients had a more pro-inflammatory gut microbiota profile with several opportunistic pathogens being enriched in patients with mild or severe disease, such as *Ba. ovatus*, *Ac. bereziniae*, *C. innocuum*, *Bu. contaminans*, and *Ba. Nordii*. In previous reports, *Ba. nordii* was found to be associated with COVID-19 [18]. Multiple virulence genes related to these species were also observed to be more abundant in severe COVID-19 patients. These microbial pathogenic factors could translocate through the leaky gut into the circulating system, promote the secretion of inflammatory cytokines by activating pattern recognition receptor-like TLRs and NOD-like receptors, and therefore lead to systemic inflammation [25]. In addition, we observed that the abundances of several microbial species changed along with the COVID-19 progression and were associated with biomarkers of host immune and inflammation. For example, *Bu. contaminans* were negatively associated with T cell-related transcription modules, which represented T cell activity and were found to reflect the dynamic immune response in COVID-19 [31]. *Bu. contaminans* was also reported in severe respiratory infection [51]. This pathogenic species could employ a type VI effector to activate the pyrin inflammasome and trigger inflammation [52], produce tyrosine kinase BceF and phosphotyrosine phosphatase BceD to strengthen the epithelial disruption, and further exacerbate the inflammation [53]. These results collectively supported a potential role of the gut microbiota in the host immune responses during COVID-19 progression.

Another potential reason for the excessive inflammation in COVID-19 patients might be the enrichment of the glycolysis pathway, which was reported to be associated with higher SRAS-Cov-2 activity [54]. Under viral and bacterial infections, especially during macrophage polarization and dendritic cell activation, the major energy metabolism switches from lipid towards glycolysis to generate ATP because of the engagement of TLR with the related activation of the PI3K/Akt pathway [55, 56]. However, the causal relationship between SARS-CoV-2 infection and the enrichment of the glycolysis pathway remains unclear, and further experimental studies are warranted.

To our knowledge, this is the first study to explore the gut microbial-host immunity crosstalk through the integration of metagenomic, proteomic, and metaproteomic approaches. These results, however, should be interpreted with caution. First, because of the case-control study design, we were not able to assess the temporal relationship of the gut microbiota with COVID-19 development. Second, residual confounding from dietary components, physical activity, BMI, and related comorbidities is possible. Third, the number of our included patients and especially the uninfected controls was relatively small, and the sample size for metaproteomic measurement was even smaller. The limited sample size influenced the statistical power of our analysis. Hence, it should be cautious when generalizing our results to other populations. Further large-scale population-based studies are warranted to validate our findings, and intervention studies could help to explore the causal roles of gut microbiota in the pathogenesis underlying COVID-19 development.

## Conclusions

In the current study, we identified several microbial features at taxonomic and functional levels that were associated with COVID-19 and its severity, as well as the host immune responses. Our result suggested that dysbiosis of the gut microbiome and the dysfunction of gut barrier might play a role in the progression of COVID-19. These findings may help identify therapeutic microbial targets that hold the potential for applications in the clinical practice of COVID-19 treatment.

## Supplementary Information

**Additional file 1: Figure S1.** The COVID-19 related inflammatory biomarkers in patients at different disease stages. **Figure S2.** Timeline of disease progression and sample collection for COVID-19 patients with multiple samples. **Figure S3.** The α-diversity of the gut microbiome among all the participants. **Figure S4.** The relative abundance of *Bacteroides nordii* throughout the hospitalization of COVID-19 patients. **Figure S5.** The relative abundance of *Blautia* sp. CAF 257 throughout the hospitalization of COVID-19 patients. **Figure S6.** The relative abundance of *Burkholderia contaminans* throughout the hospitalization of COVID-19 patients. **Figure S7.** The relative abundance of *Bifidobacterium longum* throughout the hospitalization of COVID-19 patients. **Figure S8.** The associations between COVID-19 related microbial features and RNA modules indicating T cell response. **Figure S9.** The influence of oral antibiotics uses on the gut microbiome of severe COVID-19 patients. **Figure S10.** The detail of samples for multi-omics measurement. **Figure S11.** The profiles of the gut microbiome annotated by the metaproteomics approach. **Figure S12.** The significantly differential abundant human proteins in fecal samples from COVID-19 patients and controls. **Figure S13.** The circulating levels of LPS-binding protein in COVID-19 patients. **Figure S14.** The microbial taxa identified in plasma samples from COVID-19 patients and controls through the proteomic approach.

**Additional file 2: Table S1.** The basic information of COVID-19 patients. **Table S2.** The basic information of fecal samples collected from COVID-19 patients. **Table S3.** The microbial species identified from all participants. **Table S4.** The associations between microbial species and clinical traits. **Table S5.** The abundance of microbial species in COVID-19 severe patients. **Table S6.** The abundance of microbial species in participants without using antibiotics. **Table S7.** The associations between microbial pathways and clinical traits. **Table S8.** The correlation between microbial virulence factors and clinical traits. **Table S9.** The associations between circulating level of lipopolysaccharide-binding protein and clinical traits. **Table S10.** The microbial taxa identified from all plasma samples of COVID-19 patients.

## Data Availability

Raw reads of metagenomic sequencing generated during the current study can be viewed in NODE database (https://www.biosino.org/node/project/detail/OEP002590) and are available upon acceptance of the publication.

## Abbreviations

ACE2: Angiotensin-converting enzyme 2
BMI: Body mass index
COVID-19: Coronavirus disease 2019
CRP: C-reactive protein
GI: Gastrointestinal
IL: Interleukin
LBP: lipopolysaccharide-binding protein
LEfSe: LDA effect size analysis
SARS-CoV-2: Severe acute respiratory syndrome coronavirus 2.
