# Supplementary material for: Gut microbiome alterations and gut barrier dysfunction are associated with host immune homeostasis in COVID-19 patients
Source: BMC Med. 2022 Jan 20;20:24. doi: 10.1186/s12916-021-02212-0 (PMC8769945; doi:10.1186/s12916-021-02212-0)

The gut microbiome and host immune homeostasis in COVID-19

Zhonghan Sun[1†], Zhi-Gang Song[2†], Chenglin Liu[1†], Shishang Tan[1†], Shuchun Lin[1], Jiajun Zhu[1], Fa-Hui Dai[2], Jian Gao[1], Jia-Lei She[2], Zhendong Mei[1], Tao Lou[1], Jiao-Jiao Zheng[2], Yi Liu[2], Jiang He[1], Yuanting Zheng[1], Chen Ding[1], Feng Qian[1], Yan Zheng[1*], Yan-Mei Chen[2*]

[1] State Key Laboratory of Genetic Engineering, School of Life Sciences and Human Phenome Institute, Fudan University, Shanghai, China.

[2] Shanghai Public Health Clinical Center, State Key Laboratory of Genetic Engineering, School of Life Sciences and Human Phenome Institute, Fudan University, Shanghai, China.

[*] Corresponding authors:

Yanmei Chen, Email: chenyanmei@shphc.org.cn. Shanghai Public Health Clinical Center, State Key Laboratory of Genetic Engineering, School of Life Sciences and Human Phenome Institute, Fudan University

Yan Zheng, Email: yan_zheng@fudan.edu.cn. State Key Laboratory of Genetic Engineering, School of Life Sciences and Human Phenome Institute, Fudan University, Shanghai, China.

[†] Zhonghan Sun, Zhi-Gang Song, Chenglin Liu, Shishang Tan contributed equally to this manuscript.

Supplemental figures

Fig. S1. The COVID-19 related inflammatory biomarkers in patients at different disease stages. The samples in Severe to Mild group were collected from COVID-19 patients after recovering from severe condition to mild condition.

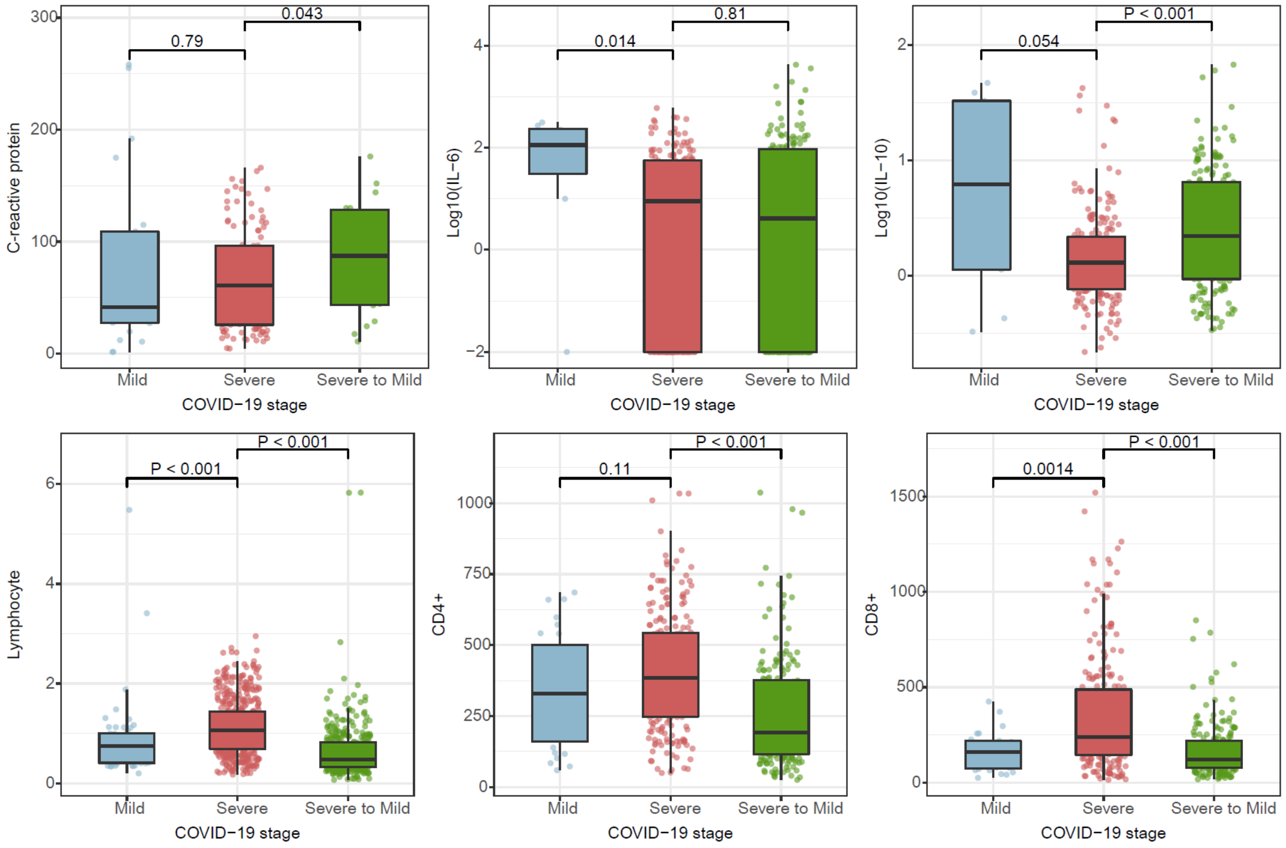

Fig. S2. Timeline of disease progression and sample collection for COVID-19 patients with multiple samples. Colored segments of the line represent different disease statuses during the hospitalization of each patient. Dotted lines represent the period of oral antibiotics use. The capitals of E with different represent the beginning (red) and ending (blue) of extracorporeal membrane oxygenator use. Pink arrows represent the incident of the cytokine storm. Yellow dots represent the time point of fecal sample collection.

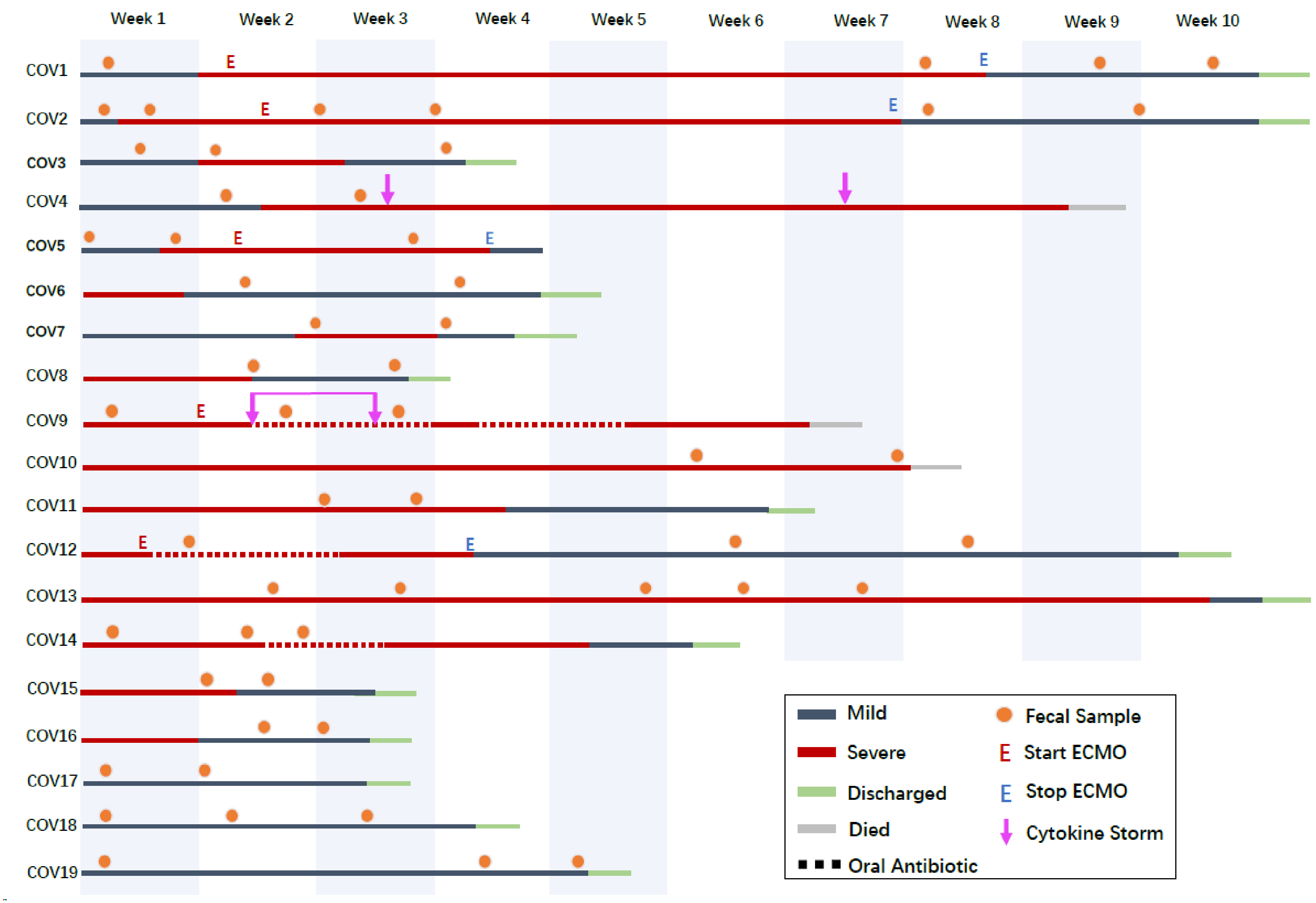

Fig. S3. The α-diversity of the gut microbiome among all the participants.

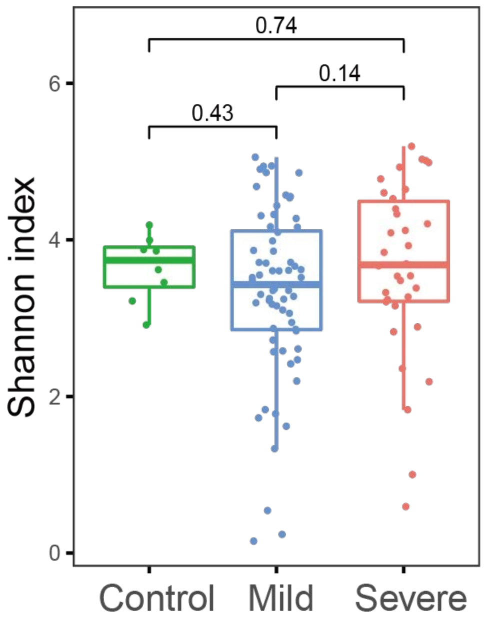

Fig. S4. The relative abundance of *Bacteroides nordii* throughout the hospitalization of COVID-19 patients. The color and shape of each time point represent the different status of COVID-19 and oral antibiotics use.

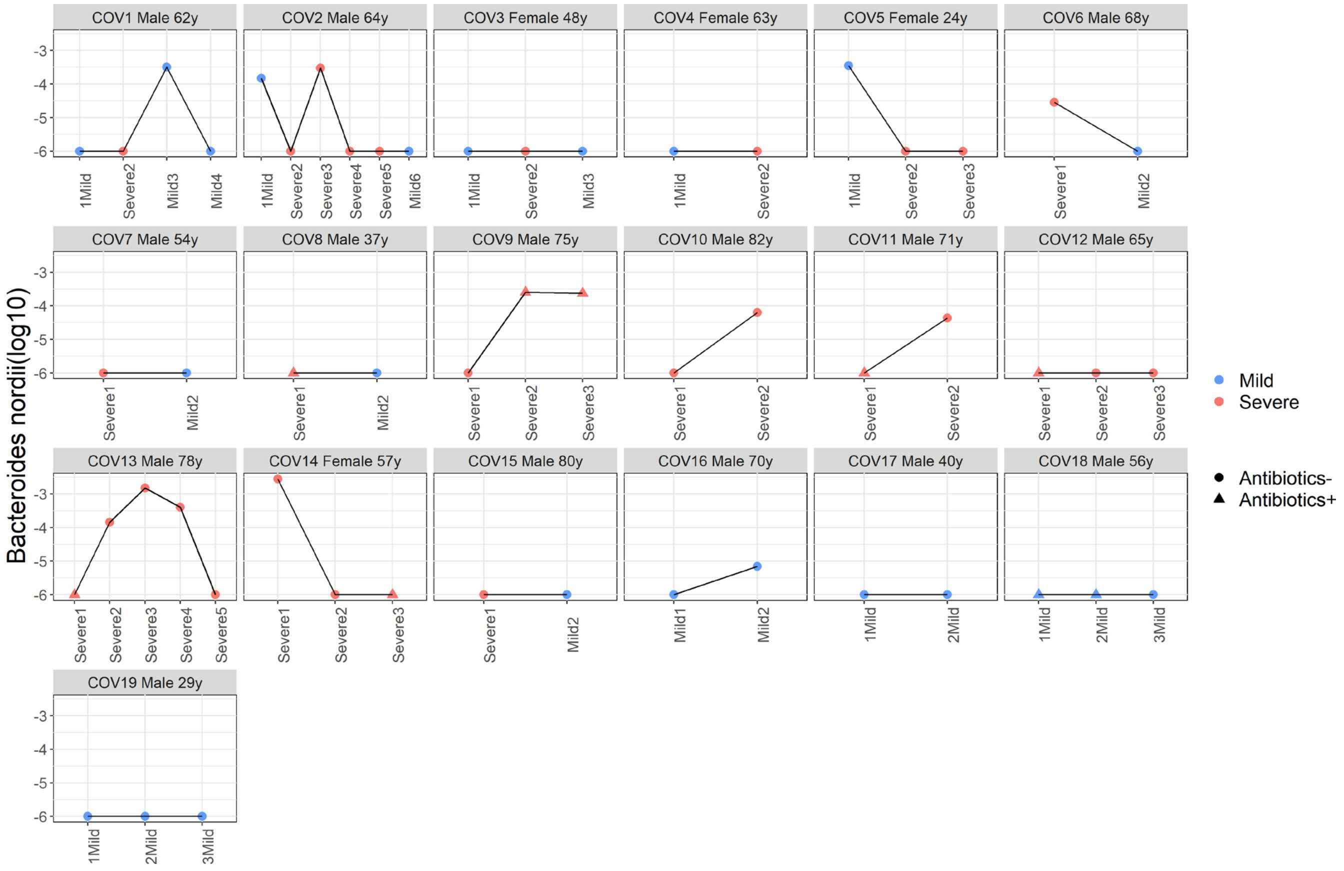

Fig. S5. The relative abundance of *Blautia sp CAF 257* throughout the hospitalization of COVID-19 patients. The color and shape of each time point represent the different status of COVID-19 and oral antibiotics use.

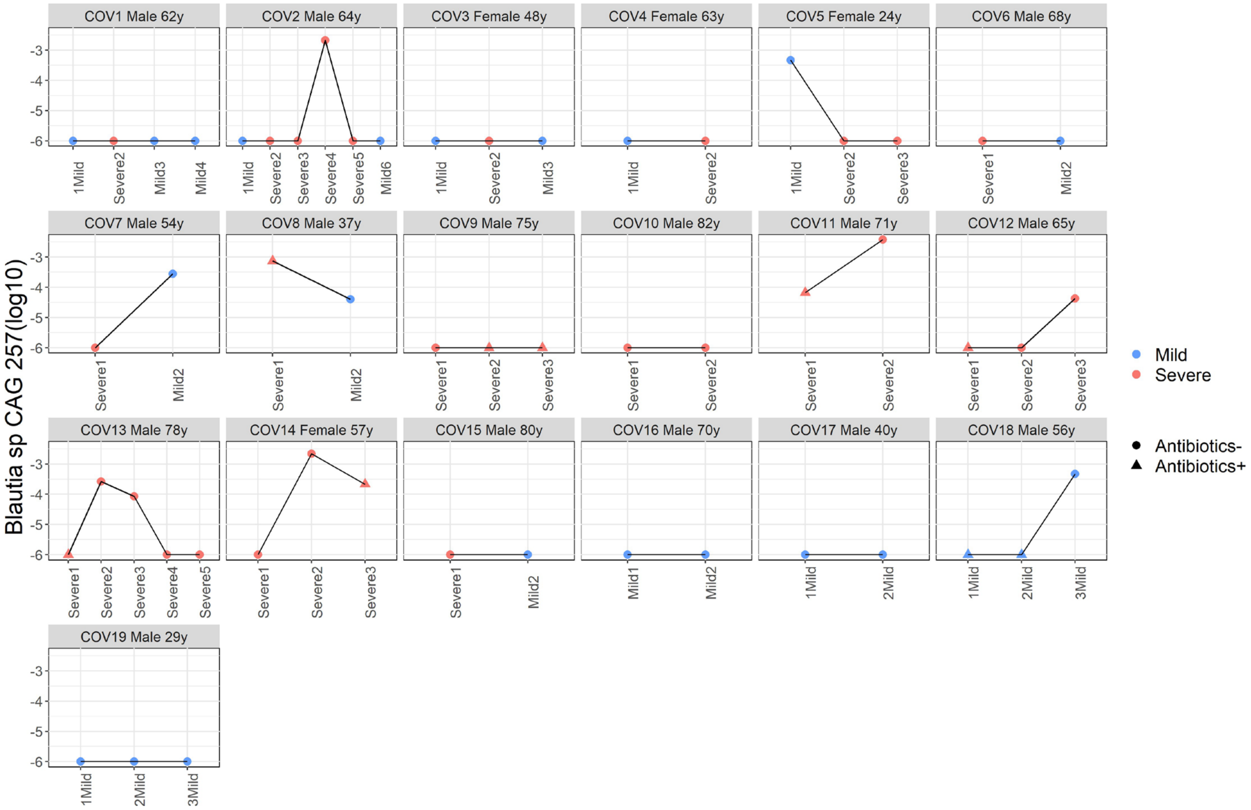

Fig. S6. The relative abundance of *Burkholderia contaminans* throughout the hospitalization of COVID-19 patients. The color and shape of each time point represent the different status of COVID-19 and oral antibiotics use.

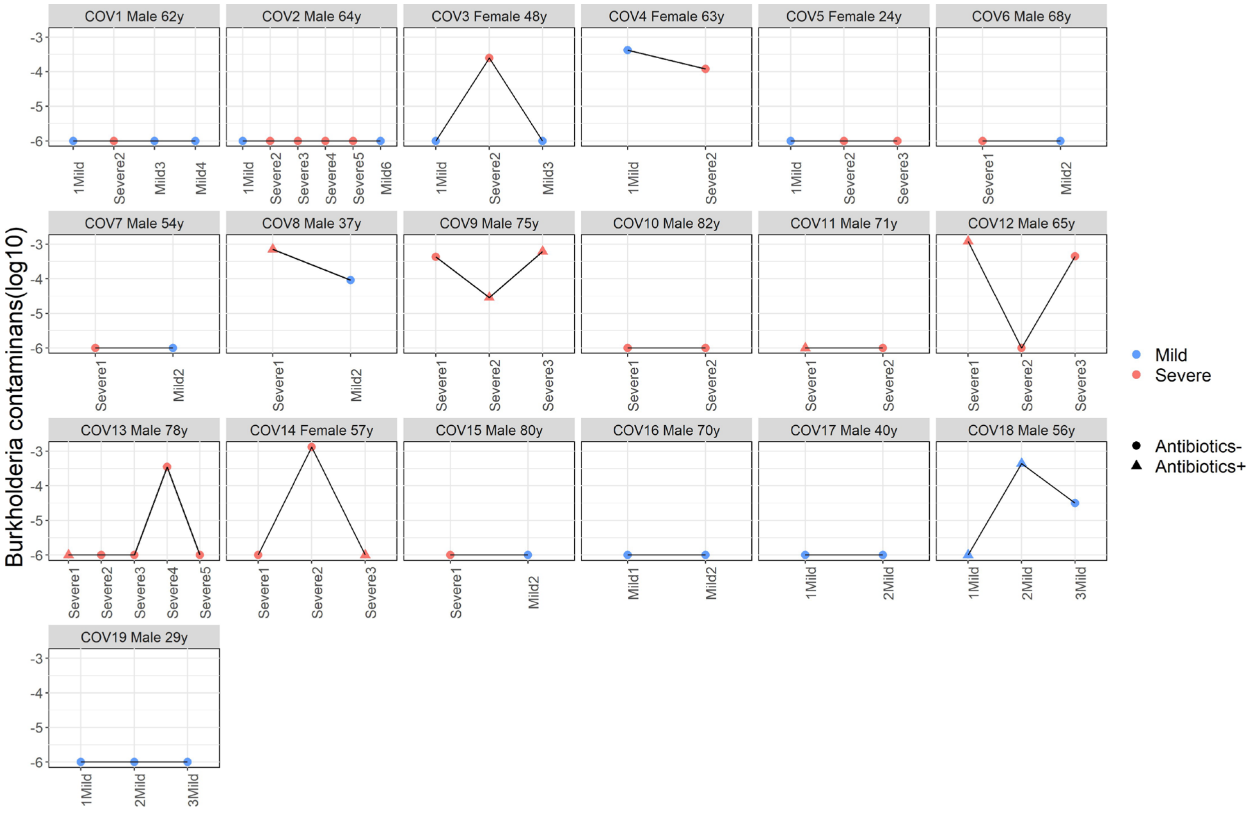

Fig. S7. The relative abundance of *Bifidobacterium longum* throughout the hospitalization of COVID-19 patients. The color and shape of each time point represent the different status of COVID-19 and oral antibiotics use.

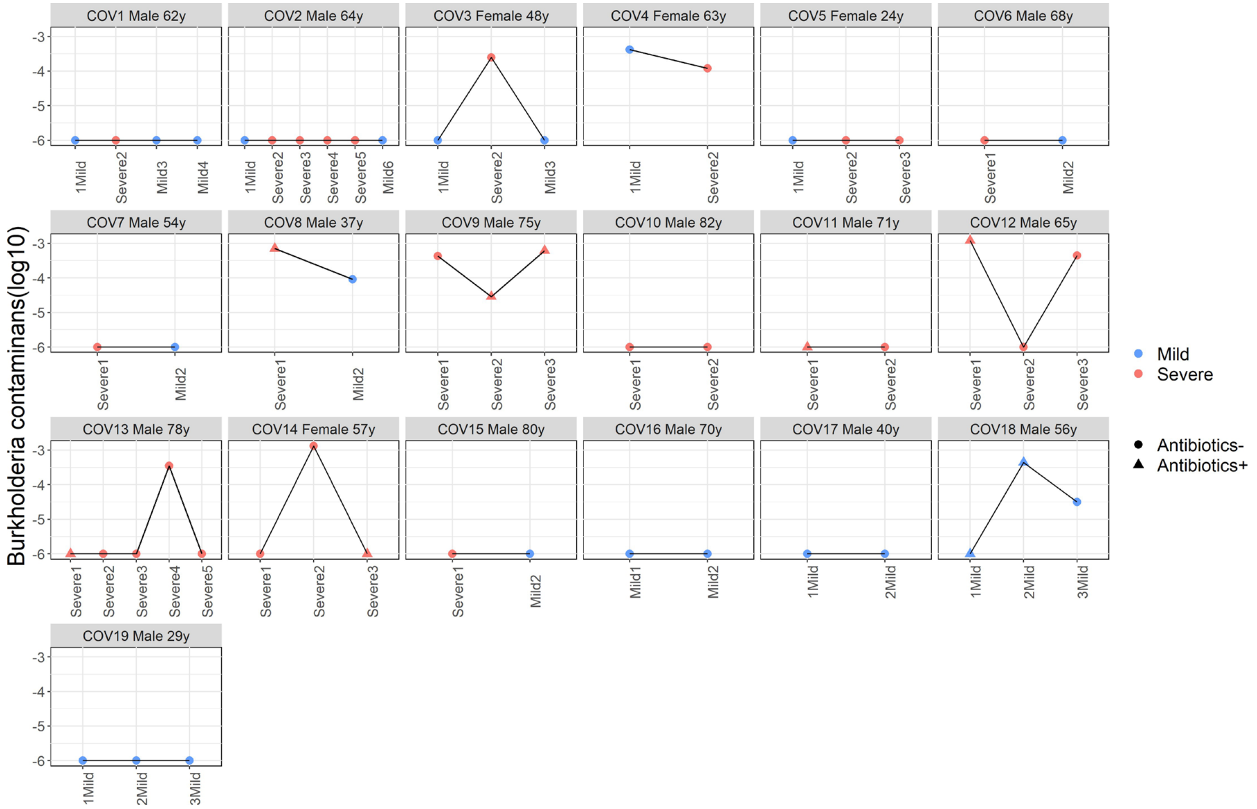

Fig. S8. The associations between COVID-19 related microbial features and RNA modules indicating T cell response.

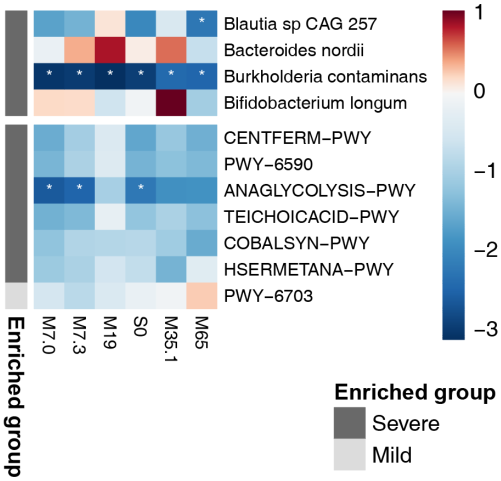

Fig. S9. The influence of oral antibiotics uses on the gut microbiome of severe COVID-19 patients. a The α-diversity of gut microbiome among severe COVID-19 patients stratified by the use of oral antibiotics. **b** to **c** The β-diversity based on Bray-Curtis dissimilarity (**b**) and Unweighted unifrac distance (**c**) at metagenomic species-level among severe COVID-19 patients stratified by the use of oral antibiotics.

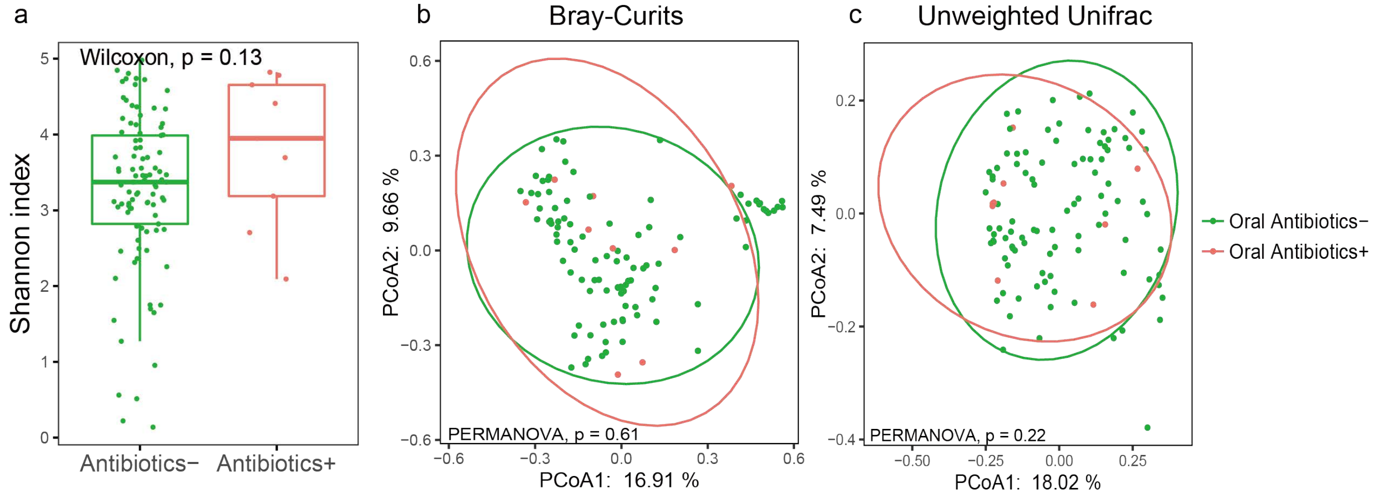

Fig. S10. The detail of samples for multi-omics measurement. Each column represents a person (patients or healthy controls). Each row represents the sample type. Each vertical bar represents the number of samples, ranging from 0 (white) to 6 (deep red). Disease status is showed on the top. A patients in mild gourp indicates this person only has mild samples, and a person in severe group indicates this person has at least one severe sample.

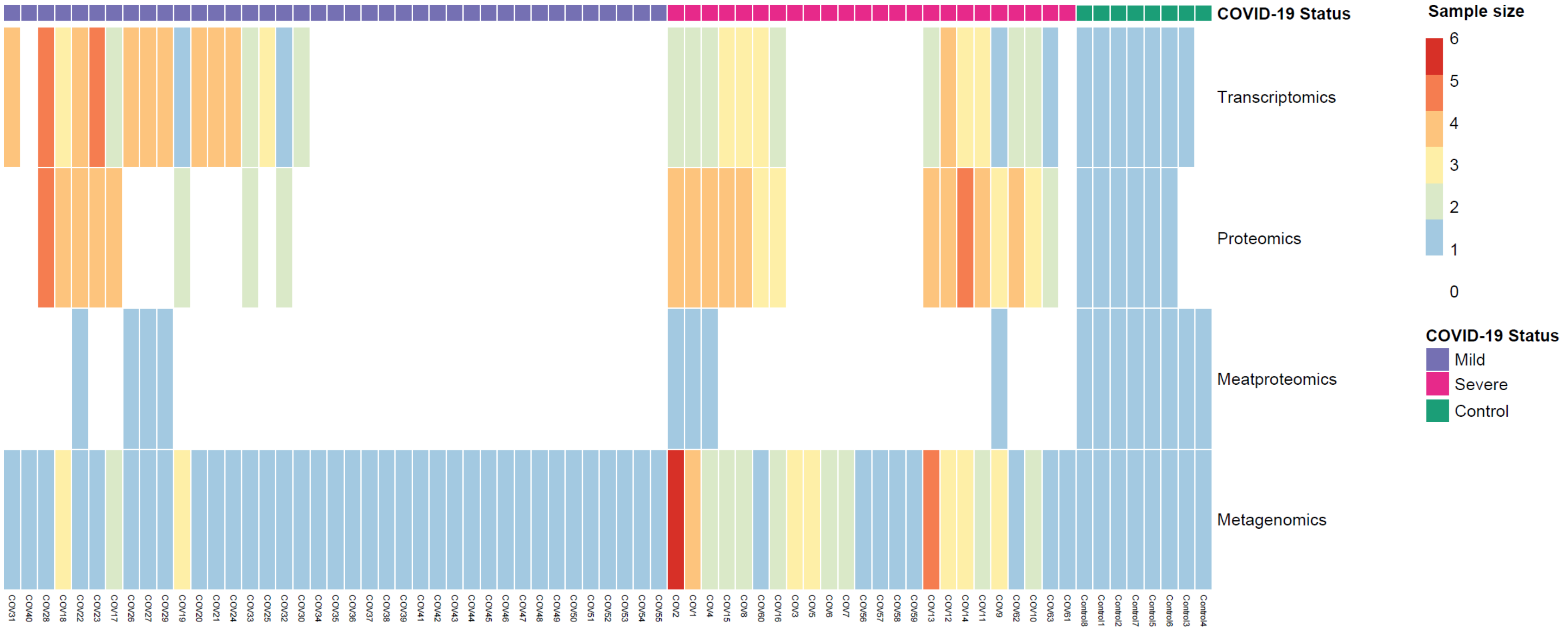

Fig. S11. The profiles of the gut microbiome annotated by the metaproteomics approach. The relative abundances of the top 5 phylum, top 10 genus, and top 20 species were shown.

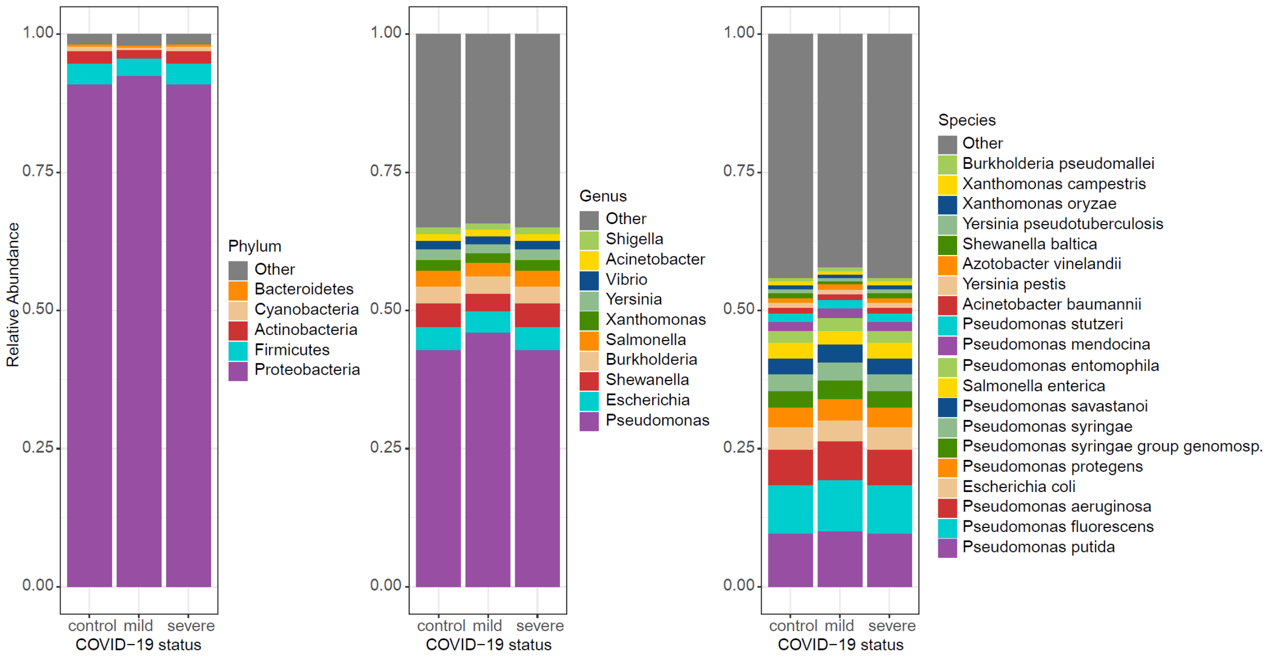

Fig. S12. The significantly differential abundant human proteins in fecal samples from COVID-19 patients and controls. Y axis indicated the mean protein abundance index within groups (PAI). The same proteins are linked between groups.

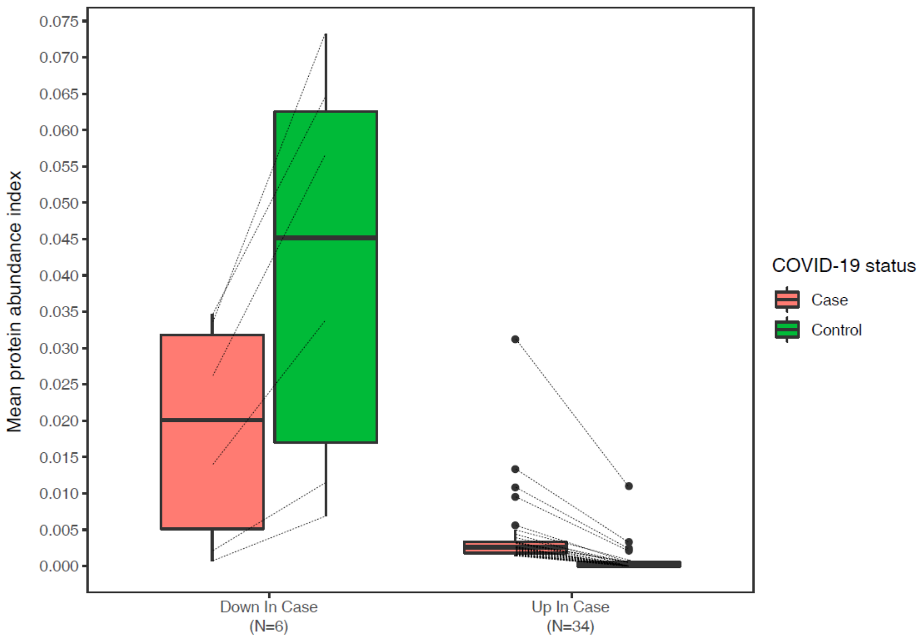

Fig. S13. The circulating levels of LPS-binding protein in COVID-19 patients.

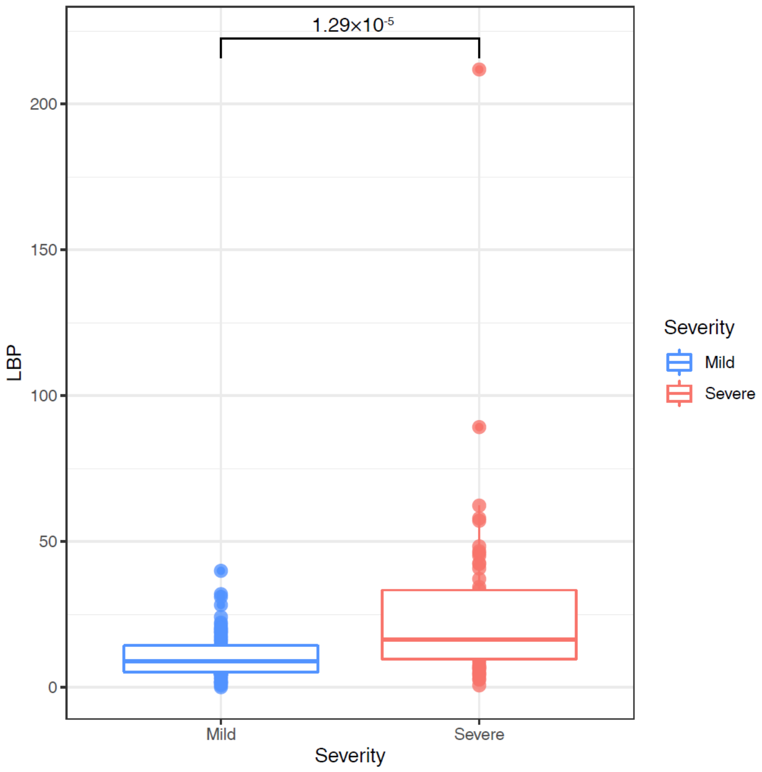

Fig. S14. The microbial taxa identified in plasma samples from COVID-19 patients and controls through the proteomic approach. Taxa with red names were detected in both fecal samples and blood samples.

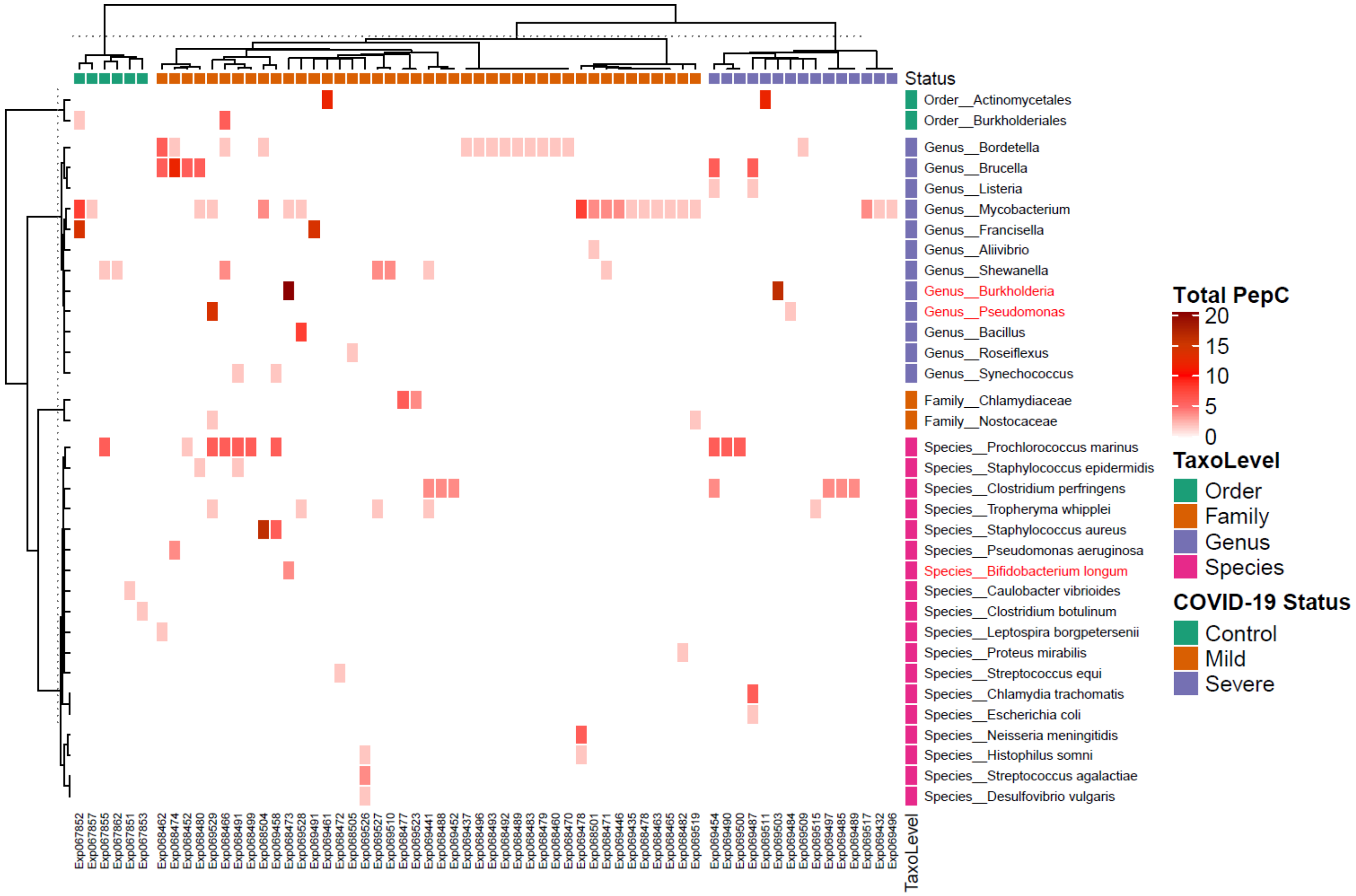

Supplement: Supplementary file 1 — Additional file 1: Figure S1. The COVID-19 related inflammatory biomarkers in patients at different disease stages. Figure S2. Timeline of disease progression and sample collection for COVID-19 patients with multiple samples. Figure S3. The α-diversity of the gut microbiome among all the participants. Figure S4. The relative abundance of Bacteroides nordii throughout the hospitalization of COVID-19 patients. Figure S5. The relative abundance of Blautia sp. CAF 257 throughout the hospitalization of COVID-19 patients. Figure S6. The relative abundance of Burkholderia contaminans throughout the hospitalization of COVID-19 patients. Figure S7. The relative abundance of Bifidobacterium longum throughout the hospitalization of COVID-19 patients. Figure S8. The associations between COVID-19 related microbial features and RNA modules indicating T cell response. Figure S9. The influence of oral antibiotics uses on the gut microbiome of severe COVID-19 patients. Figure S10. The detail of samples for multi-omics measurement. Figure S11. The profiles of the gut microbiome annotated by the metaproteomics approach. Figure S12. The significantly differential abundant human proteins in fecal samples from COVID-19 patients and controls. Figure S13. The circulating levels of LPS-binding protein in COVID-19 patients. Figure S14. The microbial taxa identified in plasma samples from COVID-19 patients and controls through the proteomic approach. [file 12916_2021_2212_MOESM1_ESM.pdf]
